# Real-World Effectiveness of Portable Air Cleaners in Reducing Home Particulate Matter Concentrations

**DOI:** 10.4209/aaqr.230202

**Published:** 2023-11-22

**Authors:** Frederic T. Lu, Robert J. Laumbach, Alicia Legard, Nirmala T. Myers, Kathleen G. Black, Pamela Ohman-Strickland, Shahnaz Alimokhtari, Adriana de Resende, Leonardo Calderón, Gediminas Mainelis, Howard M. Kipen

**Affiliations:** 1 Environmental and Occupational Health Sciences Institute, Rutgers University, Piscataway, NJ, USA; 2 Department of Environmental Sciences, School of Environmental and Biological Sciences, Rutgers University, New Brunswick, NJ, USA; 3 Department of Environmental and Occupational Health and Justice, School of Public Health, Rutgers University, Piscataway, NJ, USA; 4 Department of Biostatistics and Epidemiology, School of Public Health, Rutgers University, Piscataway, NJ, USA

**Keywords:** Particulate matter, Portable air cleaner, Filtration effectiveness, Indoor air, Naturalistic study

## Abstract

Portable air cleaners (PACs) equipped with HEPA filters are gaining attention as cost-effective means of decreasing indoor particulate matter (PM) air pollutants and airborne viruses. However, the performance of PACs in naturalistic settings and spaces beyond the room containing the PAC is not well characterized. We conducted a single-blinded randomized cross-over interventional study between November 2020 and May 2021 in the homes of adults who tested positive for COVID-19. The intervention was air filtration with PAC operated with the HEPA filter set installed (“filter” condition) versus removed (“sham” condition, i.e., control). Sampling was performed in 29 homes for two consecutive 24-hour periods in the primary room (containing the PAC) and a secondary room. PAC effectiveness, calculated as reductions in overall mean PM_2.5_ and PM_10_ concentrations during the filter condition, were for the primary rooms 78.8% and 63.9% (n = 23), respectively, and for the secondary rooms 57.9% and 60.4% (n = 22), respectively. When a central air handler (CAH) was reported to be in use, filter-associated reductions of PM were statistically significant during the day (06:00–22:00) and night (22:01–05:59) in the primary rooms but only during the day in the secondary rooms. Our study adds to the literature evaluating the real-world effects of PACs on a secondary room and considering the impact of central air systems on PAC performance.

## 1 INTRODUCTION

Air pollution, including particulate matter (PM) of various size fractions, is known to cause various adverse health effects (U.S. EPA, 2021, 2018, 2016; WHO, 2006). Exposure to PM with an aerodynamic diameter of ≤ 2.5 μm (PM_2.5_) has been associated with chronic obstructive pulmonary disease (COPD), acute and chronic cardiovascular disease, several cancers, diabetes mellitus, and dementia (Newman et al., 2020). Notably, 99% of the world population live in areas where ambient PM_2.5_ concentrations exceed the current WHO guideline for annual average concentration of 5 μg m^−3^ (WHO, 2022). For 2015, the Global Burden of Disease Study ranked PM_2.5_ exposure fifth among mortality risk factors, causing 4.2 million (95% CI: 3.7–4.8 million) deaths and 103.1 million (95% CI: 90.8–115.1 million) disability-adjusted life-years (DALYs) worldwide (Cohen et al., 2017), while the Global Exposure Mortality Model predicted 8.9 million (95% CI: 7.5–10.3 million) deaths from PM_2.5_ (Burnett et al., 2018).

Cardiovascular diseases such as myocardial infarction and stroke account for over half of deaths caused by PM_2.5_ (Rajagopalan et al., 2020). As of 2023, the largest study to date of exposure to PM and mortality examined 59.6 million deaths across 652 cities worldwide through the Multi-City Multi-Country Collaborative Research Network and reported pooled concentration-response curves that showed independent, positive associations between exposure to PM as short as 2 days (based on moving concentration average) and daily all-cause, cardiovascular, and respiratory mortality, even after adjustment for gaseous pollutants (Liu et al., 2019). Pooled daily all-cause mortality increased by 0.68% (95% CI: 0.59%–0.77%) and 0.44% (95% CI: 0.39%–0.50%) for every increase of 10 μg m^3^ in PM_2.5_ and PM_10_ (aerodynamic particle diameter ≤ 10 μm), respectively (Liu et al., 2019).

In the US, in addition to the health risks to the overall population, the exposure levels to PM_2.5_ and the resultant mortality risk are higher for racial and ethnic minorities and lower-income groups (Jbaily et al., 2022). These disparities have increased with time, underscoring the need for targeted interventions to reduce PM_2.5_ exposure in disadvantaged populations (Kaufman et al., 2020; Bowe et al., 2019). Among the common interventions to reduce our exposure to PM indoors are portable air cleaners (PACs), which target PM using filters, electrostatic precipitators, ion generators, or a combination thereof (Burton, 2006). PACs equipped with filters are generally inexpensive, practical, and known to reduce indoor PM_2.5_ levels by typically 50–60% in experimental interventions, though reported reductions may vary from 23% to 92% (Allen and Barn, 2020; Cheek et al., 2021; Maestas et al., 2019; Morishita et al., 2018). Filter-based PACs often feature high-efficiency particulate air (HEPA) filters, which capture ≥ 99.97% of particles measuring 0.3 μm in aerodynamic diameter, the most penetrating particle size (ASHRAE, 2021). In a 15 × 15 × 8 ft (1800 ft^3^) room, a typical PAC with a clean air delivery rate (CADR) of 300 cfm provides clean air equivalent to 10 air changes per hour (ACH) (Allen and Ibrahim, 2021). In contrast, ventilation-based ACH in US homes without PACs is approximately 0.5–1.5 (Reichman et al., 2017). Thus, the use of effective PACs could be instrumental in reducing PM exposures in residential environments.

Studies have evaluated the effectiveness of PACs in residential settings in reducing the indoor concentrations of various aerosol particles, including PM_2.5_, PM_10_, black carbon, and fungal spores from sources such as traffic, cooking, wildfires, and others (Barn et al., 2008; Cox et al., 2018; U.S. EPA, 2018; Sharma and Balasubramanian, 2020; Xiang et al., 2021). Since aerosol particles also play an important role in the transmission and pathogenesis of infectious diseases, such as COVID-19 or influenza (Liu et al., 2020; Santarpia et al., 2020; Zhou et al., 2021), control of exposures to indoor PM via filtration is likely to reduce exposures and transmission of viral particles indoors (Myers et al., 2022).

The advertised performance of PACs is usually based on chamber studies using well-characterized challenge aerosol particles and controlled environments. Likewise, considerations for the real-world performance of PACs, such as their impact on multiple rooms or interaction with air-conditioning systems, have also been limited to computer simulations, chamber studies, and/or experimental settings without participants, leading to a relative lack of real-world performance data (Novoselac and Siegel, 2009; Zhang et al., 2010). This knowledge gap is important to resolve because PACs in the real world are challenged by the variety of airborne particles from different sources and by air movement and ventilation conditions that are difficult to adequately simulate. While the data about the PAC performance in residential environments are limited in general, the data on a PAC’s ability to reduce PM levels outside of the room with the PAC are especially limited (Wheeler et al., 2014; U.S. EPA, 2018). To fill this knowledge gap, the first naturalistic interventional study using PACs with HEPA filters to reduce airborne levels of SARS-CoV-2 viral particles in US residences was recently completed (Myers et al., 2022; Laumbach et al., 2022). As part of the study, we also investigated the performance of PACs in reducing levels of airborne PM_2.5_ and PM_10_ in 26 homes, and the results are reported in this paper. Innovations in this study include assessing the filtration effects of PACs outside the room containing it, considering the impact of a central air handler (CAH) on PAC performance, and using a short time frame (i.e., 24 hr with a filter and 24 hr without, in a random order). We also employed low-cost PM monitors, which in recent years have gained increasing acceptance in the air quality literature (He et al., 2021; Giordano et al., 2021; Sankhyan et al., 2022).

## 2 MATERIALS AND METHODS

The details of the study design have been previously published, and here we describe only its main features (Myers et al., 2022; Laumbach et al., 2022). The study was approved by the Rutgers University Institutional Review Board (Pro2020001323), and the participants gave informed consent.

### 2.1 Study Design

Our study was a randomized cross-over trial conducted in the homes of participants in New Jersey who had recently tested positive for SARS-CoV-2. The study occurred between November 2020 and May 2021. The intervention was air filtration to reduce PM concentrations using PACs equipped with HEPA filters. A single PAC was placed in the “primary room,” which the participants chose as the space where they intended to spend most of their time during self-isolation for COVID-19 (typically a bedroom). A “secondary room” was identified as any other room in the home (most often a living or dining room). To determine the effectiveness of PACs in removing PM, the PAC was operated with the HEPA filter set installed (“filter” condition) or removed (“sham” condition) for two consecutive 24-hour periods, in randomized order. Participants were blinded to the condition (filter or sham) and were asked to operate the PAC continuously. Consistent with naturalistic study design, participants were not given any instructions or suggestions regarding behavioral patterns, including isolating for COVID-19, cooking, cleaning, or other in-home activities. We measured PM concentrations continuously in the primary and secondary rooms during the two consecutive 24-hour filter and sham conditions (~48 hours total). Room dimensions were obtained to calculate room volumes and to generate floor plans using Magicplan v9.1.2 (Sensopia, Inc., 2011–2018).

### 2.2 Participants

Participants were adults (age ≥ 18 years) who tested positive for SARS-CoV-2 infection within 7 days prior to recruitment (Myers et al., 2022). They were recruited through Rutgers University Employee Health Services (New Jersey, USA) and Vault Health (New York City, New York, USA), a virtual healthcare platform, and their positive COVID-19 status was confirmed on Day 1 based on a Rutgers-developed saliva test.

### 2.3 Participant Questionnaires

All participants completed three interviews with a sampling team member at 0 hours, 24 hours (Day 1), and 48 hours (Day 2) into field measurements. For the first interview, participants provided demographic information, the type of residence, presence of a central air handler (CAH), choice of primary and secondary rooms, and the total number of residents in the home (Table 1). At the end of each sampling day, a questionnaire was administered to document participant activity in the preceding 24 hours, including the number of hours the participant spent in the primary and secondary rooms, whether the CAH was used, and door and window-opening behaviors. We did not record information on floor coverings, cooking appliances, overhead ceiling fans, or locations of CAH intake and exhaust ports, as the investigation of the indoor air pollution sources and sinks was outside the scope of this study.

### 2.4 Portable Air Cleaner

A PAC model MA-40 (Medify Air, Deerfield Beach, Florida, USA) was placed only in the primary room about 1 meter away from the walls and any obstructions. Aware that variations in PAC location may affect overall particle removal as reported by Novoselac and Siegel (2009), we placed our PACs as consistently as possible across all homes in regard to their distances from walls and obstructions. The MA-40 model uses an integrated filter set consisting of a pre-filter, a HEPA filter, and an activated carbon filter. It is advertised to achieve a CADR of 330 cfm (9344 L min^−1^) at the highest fan setting (Mode 3). We corroborated this by using an anemometer (TSI Inc., Shoreview, Minnesota, USA) to measure the PAC’s average exit air velocity, which corresponded to a calculated air flow of 348 cfm. The quieter Mode 1, with our measured CADR of 263 cfm (no advertised rate from the manufacturer for this setting), was chosen for the study to increase the likelihood of participant adherence to continuous operation of the PAC due to lower noise level. At 1 meter from a PAC operating in Mode 1, sound levels of 59.6 ± 11.0 dB (i.e., ~13.7 dB over background levels) were measured using the NIOSH sound level meter app. The MA-40 model includes an anion generator, but we turned it off in our study.

Once used, the filter sets were retrieved and placed in cold storage for potential future studies. At the end of testing, the PAC and a replacement filter set were offered to the participants free of charge.

### 2.5 Monitoring of PM Levels

The concentrations of PM_2.5_ and PM_10_ were measured for two consecutive 24-hour periods (Day 1 and Day 2) by low-cost air quality monitors (AirVisual Pro, IQAir, Goldach, Switzerland) that employ active air sampling. Two AirVisual Pro nodes were placed in the primary and the secondary rooms at least 2 meters away from headboards, vents, windows, and obstructing furniture. The instrument estimates PM_2.5_ and PM_10_ mass concentrations using a proprietary AVPM25b sensor to detect laser light scattering caused by the laser-particle interaction (IQAir, 2022). Data are provided as PM concentrations μg m^−3^). It also measures temperature, relative humidity, and carbon dioxide levels for automated adjustment of its sensor readings. A built-in air mover provides a constant airflow through the sensing chamber. Each node was set to “Continuous Mode” to measure PM concentration every five minutes for each room during the two-day study period.

Performance of the AirVisual Pro against reference standard monitors as defined by federal equivalent methods (FEM) has been evaluated by the Air Quality Sensor Performance Evaluation Center at PM_2.5_ concentrations ranging from ~10 μg m^−3^ to ~300 μg m^−3^ (AQ-SPEC, 2018). In laboratory testing of 5-minute averaged PM_2.5_ concentrations at steady state, AirVisual Pro had *R*^2^ = 0.99 compared to an FEM instrument (Model 1.108, Environmental Dust Monitor, Grimm Aerosol Technik, Ainring, Germany). In field testing of 1-hour averaged PM_2.5_ concentrations, AirVisual Pro had *R*^2^ = 0.8236 compared to another FEM instrument (Model 1020, Beta Attenuation Monitor, Met One Instruments, Inc., Grants Pass, Oregon, USA). For field testing, the reported linear regression model was y = 0.99x + 5.2743. In both settings, intra-model variability was low, and precision was high.

While PM concentration can affect the performance of air quality monitors, this was not observed in our previously published chamber study of AirVisual Pro with varying concentrations of polystyrene latex spheres to represent monodisperse particles and Arizona Road Dust to represent environmental mineral dust (He et al., 2021). Our earlier testing also confirmed a good PM_2.5_ concentration match between three AirVisual Pro units and a DustTrak DRX (Model 8534, TSI, Inc., Shoreview, Minnesota, USA) that served as the reference device (He et al., 2021). Due to high correlation between the measurements by AirVisual Pro and reference instruments (*R*^2^ > 0.99), as reported by AQ-SPEC (2018) and us, we did not apply a correction factor in the present study. The lowest reported concentration value by the instrument is 1 μg m^−3^; PM concentrations lower than that are reported as 0 μg m^−3^.

### 2.6 Data Analysis

Study data were securely collected and managed using REDCap electronic data capture tools hosted at Rutgers Robert Wood Johnson Medical School (Harris et al., 2009, 2019). SPSS v27.0 (IBM, Armonk, New York, USA) was used to analyze and illustrate the data. The time from each 24-hour condition (filter or sham) was categorized into three periods: 24 hours (overall), 06:00 to 22:00 (“day”), and 22:01 to 05:59 (“night”). The division of day and night was intended to approximate when participants were awake and asleep, respectively, and allowed us to examine whether the performance of the PACs varied between day, when particles are likely generated during typical activities, and night, when particle generation may be reduced or is minimal. However, to avoid influencing the behavior of participants, we refrained from asking them to engage or not engage in any particular activities or to document their specific activities. PM data were stratified by the PAC’s operating condition (filter or sham), the room type (primary or secondary), the time period (overall, day, or night), and whether a CAH was used or not.

PAC effectiveness was defined as the percent difference in mean PM concentrations between sham and filter conditions:

(1) $$\text{Effectiveness}(\%):[({\mathrm{sham PM}}_{\text{x}}-{\mathrm{filter PM}}_{\text{x}})/{\mathrm{sham PM}}_{\text{x}}]\times 100\%$$

We used two approaches to assess PAC effectiveness. The first approach examined PAC effectiveness using the mean PM concentration pooled from all participants (i.e., “pooled approach”) and compared the change in the overall mean PM concentration when the PACs were operated with filters versus sham. The second approach used the mean PM concentrations from each participant’s home and intended to assess PAC effectiveness for the individual pairs of filter and sham periods (i.e., “paired approach”) for each home. However, because effectiveness is expressed as percent in Eq. (1), it is more susceptible to the effects of an absolute change in PM level in homes with low mean concentrations versus homes with high mean concentrations. Even small increases in PM concentration during sham condition compared to filter condition could result in negative effectiveness values, especially for low PM concentrations. Therefore, homes were divided into those with low mean PM concentrations (“low-PM,” i.e., ≤ 5 μg m^3^) and high mean PM concentrations (“high-PM,” i.e., > 5 μg m^−3^) as measured in the primary room when the PAC was in sham operating condition.

Summary statistics of observed PM concentrations in μg m^−3^ in each home were expressed as mean ± standard deviation (SD) and median values for the filter and sham periods. The effects of PACs on PM_2.5_ and PM_10_ concentrations stratified by room type, time period, and usage of a CAH were examined. For data analysis, a CAH was considered in use if a participant reported its activation for any length of time during this 48-hr study. It was considered not in use if absent from the home or reported as inactive during this study.

The statistical significance of differences in mean PM concentrations between filter and sham conditions was analyzed by a paired t-test. However, if the PM data had a non-normal distribution based on the Shapiro-Wilk test, a Wilcoxon signed-rank test (WSRT) was used to compare median PM concentrations between filter and sham conditions. To compare PAC effectiveness values for PM_2.5_ and PM_10_ between high-PM homes and low-PM homes, either a paired t-test or a WSRT was used, depending on whether PAC effectiveness data were normally distributed as per the Shapiro-Wilk test. To compare PAC effectiveness values for PM_2.5_ and PM_10_ between homes with a CAH in use and homes with no CAH in use, which are independent groups, either an unpaired t-test or a Mann-Whitney *U* test (MWUT) was used, depending on whether PAC effectiveness data were normally as per the Shapiro-Wilk test.

Significance level was set at *p*-value < 0.05. Results with borderline statistical significance (*p* < 0.1) were also reported as small sample sizes limited the power for statistical testing. Comparisons were stratified by time period and room type.

## 3 RESULTS

### 3.1 Number of Participants

**Fig. 1.**
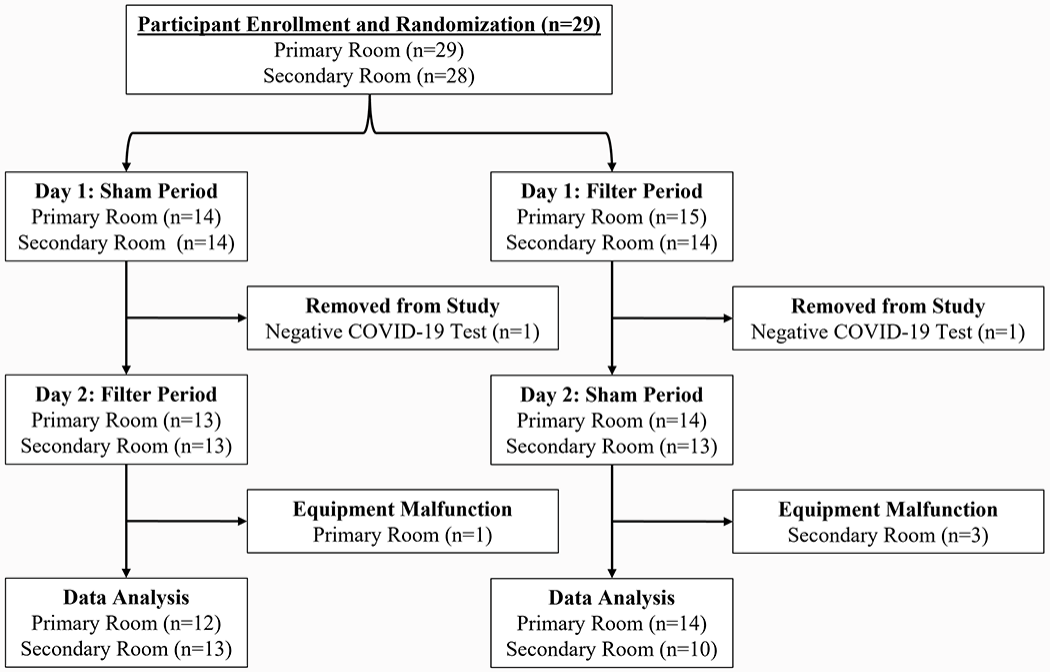
Flow diagram of the number of study participants.

Of the 29 homes enrolled, 27 homes were included in data analysis. Complete PM data for the sham and filter periods were obtained from 26 primary and 23 secondary rooms (Fig. 1). Only one home did not have a secondary room because it was a studio apartment. Two homes were excluded due to missing data from sham or filter days. Due to equipment malfunction, data from 1 primary room and 3 secondary rooms were also removed.

### 3.2 Demographics, Residence Types, Survey Data, and General Sampling Conditions

Frequency and percentages of demographic data, residence types, and questionnaire responses are presented in Tables 1 and 2. Approximately half of participants lived in single-family detached homes (n = 14, 51.9%). The remainder lived in multi-apartment townhouses (n = 7, 25.9%) and apartment buildings (n = 5, 18.5%). Twenty-two homes (81.5%) were occupied by two or more people.

The primary rooms averaged a volume of 1,858 ± 576 ft^3^, and the secondary rooms averaged a volume of 2,340 ± 1,222 ft^3^. Participants reported spending similar amounts of time (~17 hours) in the primary room during filter and sham conditions. When the study participants did not use the primary rooms, the doors to the primary rooms were kept closed by slightly fewer than half of participants during filter (13/27, 48%) and sham (12/27, 44%) conditions, respectively.

Because sampling was performed between November 2020 and May 2021, when average outdoor temperatures ranged from 36.7°F (2.6°C) to 56.8°F (13.8°C) (NCEI, 2023), the heating was generally on in participants’ homes, of which 63.0% had forced air (i.e., a central air handler, or CAH), 25.9% had hot water radiator/baseboards, and 11.1% had electric heating systems. Most participants reported windows always closed in the primary (22/27, 81.5%) and secondary (21/26, 80.8%) rooms throughout the sampling period.

### 3.3 Particulate Matter (PM) Concentrations

**Fig. 2.**
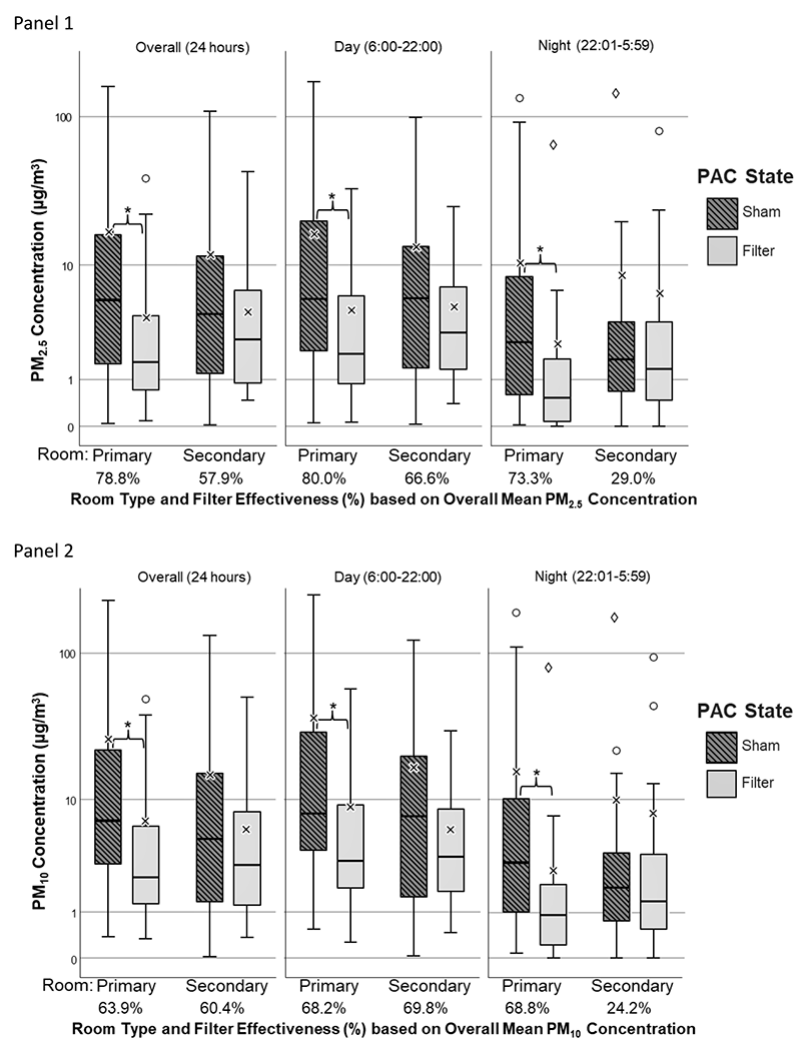
Concentrations of PM_2.5_ (Panel 1) and PM_10_ (Panel 2) as stratified by room type, time period, and PAC state; mean PM concentrations noted with “X” marks; effectiveness values from Table 3 noted on the x-axis; * for *p* < 0.05, as per WSRT. Outliers: diamond (◇) for greater than Q3 + 3 IQRs or less than Q1 − 3 IQRs and circle (◯) for within Q3 + (1.5 to 3 IQRs) or within Q1 − (1.5 to 3 IQRs), where Q = quartile and IQR = interquartile range.

The measured mean and median concentrations of PM_2.5_ and PM_10_ stratified by PAC operating condition (filter or sham) and by time period (overall, day, and night) are presented in Table 3 and Fig. 2. The mean concentrations of PM_2.5_ and PM_10_ varied considerably house to house and were highest during the day period when the PAC was in sham state, with PM_2.5_ averaging 25.4 ± 47.1 μg m^−3^ (range: 0.05 μg m^−3^ to 169.5 μg m^−3^) and PM_10_ averaging 36.6 ± 63.8 μg m^−3^ (range: 0.5 μg m^−3^ to 241.0 μg m^−3^). As the PM concentrations in individual homes were not normally distributed (statistical test data not shown), the WSRT was applied to test the differences in median PM concentrations between the filter and sham operating conditions and between the day and night periods (Table 3).

For the primary rooms, the overall mean and median concentrations of PM_2.5_ and PM_10_ decreased when the PAC was in filter state as compared to sham state, and this was consistent for overall, day, and night periods (Fig. 2). The decrease in median concentrations was significant (*p* < 0.01 for all conditions), as per WSRT. (As mentioned above, the PM data failed the normality test; thus, the mean concentrations between sham and filter conditions were not compared.) For the secondary rooms, a similar decrease in mean and median levels of PM_2.5_ and PM_10_ was observed for all time periods, albeit without reaching statistical significance (*p* > 0.05). When the open/closed state of the doors to the primary rooms and of the windows to the primary and secondary rooms was considered, the decrease in PM_2.5_ and PM_10_ concentrations did not reach statistical significance (*p* > 0.05).

### 3.4 PAC Effectiveness based on Overall PM Concentrations (Pooled Approach)

Using the pooled approach, the effectiveness of PACs in reducing mean PM_2.5_ concentrations for the overall time period was 78.8% in the primary rooms and 57.9% in the secondary rooms (Table 3). For reducing mean PM_10_ concentrations during the overall time period, PACs had an effectiveness of 63.9% in the primary rooms and 60.4% in the secondary rooms. In general, effectiveness was greater at day than at night for PM_2.5_ in both primary rooms (80.0% versus 73.3%) and secondary rooms (66.6% versus 29.0%) and for PM_10_ in secondary rooms only (69.8% versus 24.2%). The exception was for PM_10_ in primary rooms, for which PAC effectiveness during day and night was similar (68.2% versus 68.8%).

PAC effectiveness was not significantly associated with room volume, as per linear regression analysis (statistical test data not shown). Further sensitivity analyses did not find a significant association between PAC effectiveness and the open/closed state of the surveyed doors and windows (p>0.05).

### 3.5 The Role of a Central Air Handler (CAH)

The effect of a central air handler (CAH) on PM_2.5_ and PM_10_ concentrations and PAC effectiveness (%) in individual homes is shown in Table 4.

For primary rooms in homes with a CAH in use, there was a statistically significant decrease (*p* < 0.01) in median PM concentrations for all time periods when the PAC was in filter state, which may indicate a potentiating effect of CAH usage on PAC effectiveness. For primary rooms in homes with no CAH in use, the only statistically significant decrease in median PM concentration when the PAC was in filter state was for PM_10_ at night.

For secondary rooms in homes with a CAH in use, the decrease in median PM_2.5_ and PM_10_ concentrations was significant (*p* < 0.05) for the day period only. In homes with no CAH in use, secondary rooms showed non-significant decreases in mean and median levels of PM_2.5_ and PM_10_ during filter state for all time periods.

Between homes with a CAH in use and homes with no CAH in use, PAC effectiveness values did not significantly differ as per MWUT (*p* > 0.05).

### 3.6 PAC Effectiveness in Individual Homes (Paired Approach)

**Fig. 3.**
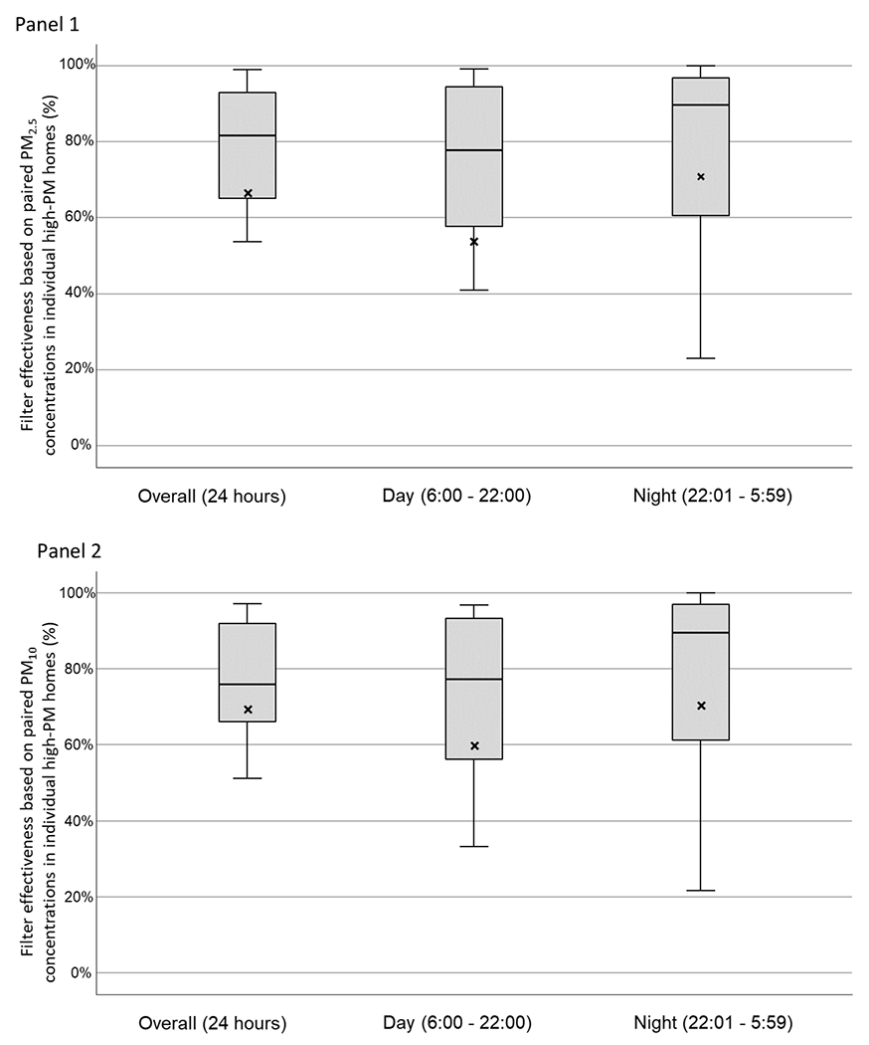
PAC effectiveness in reducing PM_2.5_ (Panel 1) and PM_10_ (Panel 2) in high-PM homes, as determined by paired approach and stratified by time period; mean PM concentrations noted with “X” marks.

Using the paired approach, the median of the individual effectiveness values for PACs in reducing mean PM_2.5_ concentrations in high-PM homes for the overall time period was 81.7% for primary rooms and 55.2% for secondary rooms (Table 5 and Fig. 3). For PM_10_ concentrations, the median of the individual PAC effectiveness values was 75.9% for primary rooms and 68.9% for secondary rooms during the overall time period. As expected, the median of the effectiveness values for the low-PM homes was sometimes negative (Table A2 in Appendix). In the high-PM homes, this was also seen in secondary rooms at night when mean PM_2.5_ and PM_10_ concentrations were lower relative to the day period (Table 5).

For both high-PM homes and low-PM homes, in the primary rooms the median of the individual PAC effectiveness values for PM_2.5_ and PM_10_ was greater at night than at day, while in secondary rooms the median of these values was lower at night than at day. However, these differences between day and night for the PAC effectiveness values for PM_2.5_ and PM_10_ did not reach statistical significance (*p* > 0.05).

## 4 DISCUSSION/CONCLUSION

### 4.1 Significance

Our naturalistic study, with no control of participants’ behavior, shows that PACs could be an effective means to reduce PM concentrations in homes. Remarkably, statistically significant reductions in PM_2.5_ and PM_10_ concentrations were observed in just a single 24-hour intervention period as compared to a sham condition of also 24 hours, with a randomized cross-over design. While our intervention period was shorter than that of other field studies on PAC effectiveness, we found similar results with these studies as discussed in the following section.

In addition to examining PM concentration reductions in the primary room, where the PAC was located, our study is among the first to assess the filtration effects of PACs on a secondary room. Our data demonstrate that a PAC can substantially reduce PM concentrations not only in the primary room but also in other rooms in a home. This is consistent with findings by Sankhyan et al. (2022) that daytime usage of a PAC in residential kitchens was associated with lower PM_2.5_ concentrations in both the kitchens and nearby bedrooms.

Similar to several other studies, our study also evaluated the impact of a central air handler (CAH) on PAC performance (Shrestha et al., 2021; Coyle et al., 2021; Zhang et al., 2010; Ward and Corsi, 2005). We acknowledge that CAH settings and operation times differ from home to home and may be influenced by building factors, residents’ socioeconomic status, and other variables. Nevertheless, even without knowing the exact duration of use of a CAH, we found that homes with any reported use of a CAH showed enhanced reductions of PM by a PAC in the primary rooms during the 24-hour intervention period and even in the secondary rooms during day intervention period when PM source emissions, such as cleaning or cooking, are more likely to occur. Operation of a CAH enhances air flow and mixing within rooms and between rooms. This consequently increases the effective CADR of the PAC. Notably, our data may show a potentiating relationship between CAHs and PACs because in homes with no CAH in use, PM concentrations even in the primary room containing the PAC did not have statistically significant reductions. This reinforces our observation and those of previous studies that a CAH is an important enhancer of air mixing that potentiates PAC filtration effectiveness. For instance, our data align with the finding by Coyle et al. (2021) that, compared to a CAH operating alone in a simulated meeting room, the addition of two PACs further reduced the mean aerosol mass concentration by 0.8% per unit increase in ACH.

### 4.2 Comparison of Data with Other Studies

Both pooled and paired data analysis approaches show PAC effectiveness in reducing PM concentrations. We found good agreement between the mean PAC effectiveness from the pooled approach and the median of PAC effectiveness values in high-PM homes from the paired approach in reducing mean PM concentrations during the overall time period (Table 3): 78.8% (pooled approach) versus 81.7% (paired approach) for PM_2.5_ in the primary rooms, 57.9% versus 55.2% for PM_2.5_ in the secondary rooms, 63.9% versus 75.9% for PM_10_ in the primary rooms, and 60.4% versus 68.9% for PM_10_ in the secondary rooms.

As mentioned, we observed PAC effectiveness in both the primary and secondary rooms over a brief 24-hr intervention period. In contrast, many previous studies of PAC filtration effectiveness used longer intervention periods. Reported mean or median effectiveness in reducing PM_2.5_ concentrations include 53.5% over 6 months (Woo et al., 2023), 54.5% over two weeks (Spilak et al., 2014), 60% over one week (Allen et al., 2011), 48.2% in mostly smoking households over one week (Weichenthal et al., 2013), and 59.8% in bedrooms and 61.2% in living areas over 48 hours (Bräuner et al., 2008). The Cardiac Rehabilitation Air Filter Trial found that personal-level PM_2.5_ exposures over 24 hours for an elderly outpatient population decreased by 43.8% with continuous use of a PAC in the bedroom and a second PAC in another main living space (Bard et al., 2020). PM levels in all these studies were determined using filter sampling and gravimetric analysis.

Our study’s reported PAC effectiveness values for reducing mean PM_2.5_ and PM_10_ concentrations in the primary rooms are similar to what has typically been reported. In terms of absolute PM levels, we found that PAC filtration reduced median PM_2.5_ and PM_10_ concentrations in the overall time period from 5.6 μg m^−3^ to 1.6 μg m^−3^ and from 7.0 μg m^−3^ to 2.4 μg m^−3^, respectively, in the primary rooms and from 4.3 μg m^−3^ to 2.7 μg m^−3^ and from 5.1 μg m^−3^ to 3.1 μg m^−3^, respectively, in the secondary rooms (Table 3). Comparable studies have typically lowered PM_2.5_ levels to approximately 4 μg m^−3^ in the room(s) containing PACs (Spilak et al., 2014; Allen et al., 2011; Bräuner et al., 2008). As one of the first studies to evaluate the effectiveness of a PAC on a secondary room, we found that our overall PAC effectiveness values of 57.9% for PM_2.5_ and 60.4% for PM_10_ for these secondary rooms largely resemble the results of the previously mentioned studies regarding overall reductions in PM_2.5_ and PM_10_ concentrations. While a longer sampling period may provide a more accurate and precise estimate of PAC effectiveness within each home, the similarities between our data and others despite our relatively brief 24-hour filtration period indicate a promising role for indoor air quality studies with shorter intervention periods. It should be added that the installed PACs were intended to reduce the transmission of SARS-CoV-2 and have likely succeeded (Myers et al., 2022).

In high-PM homes, PAC filtration was associated with larger reductions in absolute PM concentrations compared to low-PM homes. Additionally, in low-PM homes, the calculated effectiveness values within homes were often negative, most likely due to day-to-day variations in contributions by outside or indoor sources of PM. For example, cleaning or cooking during a filtration period and absence of such an activity during the corresponding sham period could result in a negative effectiveness value in a paired comparison. At low absolute PM concentrations, these factors became relatively more important as a simple matter of signal-to-noise ratio. Even with these factors, we show PAC effectiveness of close to 80% (Tables 3 and 4).

### 4.3 Limitations

The effectiveness of PACs in homes may be influenced by many factors that vary from home to home and within homes over time, such as the condition and operation of ventilation systems, when present, and the state of the homes’ building envelope/enclosure, which affects airflow and particle penetration between the indoor and outdoor environment (Liu and Nazaroff, 2001; Mudarri, 2010). Indoor appliances and furnishings associated with increased indoor PM levels included natural gas cooking appliances and carpeted floors (He et al., 2022; Branwell et al., 2016; Sagona et al., 2017; Spilak et al., 2014). However, the investigation of the effect of floor coverings, cooking appliances, overhead ceiling fans, or locations of CAH intake and exhaust ports was outside the scope of our study.

The proximity of PACs to an emission source and physical obstacles can significantly affect the mixing and transport dynamics of aerosols in the room (Küpper et al., 2019). Air mixing patterns and room volumes can influence PAC performance (U.S. EPA, 2022). In chamber experiments and at well-mixed conditions, the chamber (room) volume affects the time required to reach steady state in PM concentrations after a change in the infiltration or exfiltration rate of PM for a room and/or after an air filter is turned on or off. However, room volumes did not appear to significantly affect PAC filtration effectiveness in our study (data not shown). This is consistent with findings by Nazaroff (2004) and Spilak et al. (2014).

Our sham condition was an imperfect counterfactual as the strength of indoor PM sources, outdoor PM concentrations, air exchange rates, and other factors that determine indoor PM concentrations would be expected to vary from one 24-hour period to the other. It is likely that the variation in these factors was independent of the sham vs. filter condition to which the participants were blinded. While it was therefore unlikely to have biased our results, this variation likely decreased the precision of our estimates of PAC effectiveness and also contributed to observed negative effectiveness values in our paired approach at low PM concentrations. On the other hand, source variability represented actual conditions and changes in PM, unlike in scripted or chamber studies.

Other factors potentially affecting the comparability of our results to other studies include differences due to geographic location, weather, building design, and occupant behavior. Our study was limited to participants in New Jersey and suburban New York. Residential building codes in these locations might require relatively tighter building envelopes due to the cold and mixed-humid climate, i.e., climate zones 4–5 (Baechler et al., 2015). Additionally, our study was conducted during a period of mostly cold weather with 88.2% of participants reporting that their windows were always closed.

From a behavioral standpoint, participants did not record their specific activities, so we were not able to take these potential sources of PM into account. For the open/closed state of the door to the primary room, our survey questionnaire limited responses to categorical options (closed, closed when in room, and open). While participants self-reported their behavior regarding the opening of the windows in the primary and secondary room, our survey questionnaire elicited the number of hours, not which hours, that window(s) was/were open in each room. This precludes an examination of more specific temporal effects, if any, of window-opening behavior on PM concentration and filtration effectiveness, though any such effect in our study data is likely mitigated by most (> 80%) participants reporting windows always closed throughout the sampling period.

While all of our participants had tested positive for COVID-19, study personnel did not give them any instructions regarding self-isolation. The general expectation to self-isolate at the time of the study may have led to behavioral changes that influenced air mixing patterns and/or the generation of PM from indoor sources, in turn affecting the PAC’s filtration effectiveness. Efforts to keep the door closed in the primary room, for example, may have biased our results towards greater filter effectiveness in the primary room compared to “usual” conditions.

### 4.4 Future Directions

Future studies could consider several changes. The composition of PM particles from different homes could be assessed using elemental analysis by inductively coupled plasma mass spectrometry (ICP-MS) and other suitable methods. PAC filters could provide integrated air samples for this purpose. Particle sources and sinks may be better characterized by recording the types of floor coverings and cooking appliances as well as the locations of any CAH intake/exhaust ports and overhead ceiling fans and their use. Tracking the amount of time that room doors and windows, if present, were open and having participants keep an activity log with a level of detail down to each hour would allow better examination of the interactions between behavioral phenomena and PAC filtration effectiveness, assuming that recall bias is minimized. The advantage and convenience of do-it-yourself (DIY) air cleaners (e.g., Corsi-Rosenthal box and others) in reducing indoor PM should be further explored. Emerging data show that they could be as effective as commercially available PACs (Myers et al., 2023).

Adequately powered trials of PAC interventions with health outcomes are needed to improve our understanding of the potential health benefits of PACs. Improving household indoor environmental quality may be especially important for the increasing numbers of work-from-home employees who are spending more time in their homes and home offices.

## Supplementary Material

Appendices, including supplementary tables and figures

## Figures and Tables

**Table 1.** Demographic, residential, and survey data of the study participants.

Categories	Frequency (n = 27)	Percentage (%)
Gender		
Female	9	53
Male	8	47
Race		
Asian	4	24
Black or African American	3	18
White	7	41
Prefer not to answer	3	18
Hispanic or Latino		
Yes	3	18
No	14	82
Residence		
A single-family detached house	14	52
A townhouse or multi-family house with 2 or 3 apartments	7	26
An apartment in a building with 4 or more apartments	5	19
Other	1	3.7
HVAC system (heat generally on)		
Central air handler (CAH)	17	63
Hot water (radiator/baseboard)	7	26
Electric	3	11
Occupancy		
Two or more residents	22	82
Participant only	5	19

**Table 2.** Participant behavior and room characteristics. Results are shown as either mean ± standard deviation or count (percentage) as appropriate. Percentages not adding up to 100% are due to rounding.

Participant Behavior			

	Options	PAC Operating Condition	
		Filter	Sham
Time Spent in Room (hours)	Primary Room	16.7 ± 5.6	16.8 ± 5.7
	Secondary Room	5.4 ± 4.7	5.6 ± 5.1
Window(s) Open Time (hours)	Primary Room	1.6 ± 4.8	1.2 ± 4.6
	Secondary Room	1.1 ± 4.7	1.2 ± 2.8
Door State	Closed	13 (48%)	12 (44%)
	Closed When in Room	5 (19%)	7 (26%)
	Open	9 (33%)	8 (30%)
Room Characteristics			

	Volume (ft^3^)	Primary	Secondary
	
		1,857.6 ± 575.7	2,339.9 ± 1,222.2

Type	Bedroom	16 (59%)	8 (31%)
	Living Room	10 (37%)	15 (58%)
	Other	1 (3.7%)	3 (11.5%)
	Total	27	26*

* One home was a studio apartment with no secondary room

**Table 3.** PM concentrations and portable air cleaner (PAC) effectiveness (%) stratified by room type and time period. PAC Effectiveness = [(Mean Sham PM − Mean Filter PM)/Mean Sham PM] × 100%. WSRT = Wilcoxon-signed rank test to compare median PM values between filter and sham conditions. *p* < 0.05 was considered significant (bolded).

Room Type	PM Fraction	Time Period	n	Sham		Filter		Effectiveness (%)	WSRT p-value
				Median (μg m^−3^)	Mean ± SD (μg m^−3^)	Median (μg m^−3^)	Mean ± SD (μg m^−3^)		
Primary	PM_2.5_	Overall	26	5.55	21.17 ± 39.59	1.59	4.49 ± 8.41	78.8	0.002
		Day	26	5.64	25.41 ± 47.05	1.93	5.08 ± 8.11	80.0	0.003
		Night	26	2.49	12.84 ± 30.29	0.52	3.35 ± 12.05	73.3	< 0.001
	PM_10_	Overall	26	7.02	30.11 ± 53.45	2.40	7.31 ± 12.13	63.9	0.001
		Day	26	8.02	36.63 ± 63.75	3.38	8.91 ± 14.05	68.2	0.002
		Night	26	3.23	17.20 ± 40.60	0.92	4.19 ± 14.61	68.8	< 0.001
Secondary	PM_2.5_	Overall	23	4.30	13.44 ± 25.07	2.65	5.65 ± 8.96	57.9	0.089
		Day	23	5.78	15.62 ± 26.99	3.02	5.23 ± 5.84	66.6	0.068
		Night	23	1.70	9.24 ± 28.90	1.34	6.57 ± 16.91	29.0	0.910
	PM_10_	Overall	23	5.08	17.43 ± 31.14	3.07	6.91 ± 10.75	60.4	0.114
		Day	23	7.53	20.85 ± 34.92	3.60	6.30 ± 7.02	69.8	0.064
		Night	23	1.91	10.83 ± 34.63	1.34	8.21 ± 20.71	24.2	0.951

**Table 4.** Effect of a central air handler (CAH) on PM_2.5_ and PM_10_ concentrations and PAC effectiveness (%) in individual homes. “In Use” = activation of the CAH for any length of time; “Not in Use” = CAH not activated or not present. SD = standard deviation. WSRT = Wilcoxon signed-rank test. *p* < 0.05 was considered significant (bolded).

Room Type	PM Fraction	Time Period	Central Air Handler in Use							Central Air Handler Not in Use						
				Sham		Filter		Effectiveness (%)	WSRT *p*-value		Sham		Filter		Effectiveness (%)	WSRT *p*-value
			n	Median (μg m^−3^)	Mean ± SD (μg m^−3^)	Median (μg m^−3^)	Mean ± SD (μg m^−3^)			n	Median (μg m^−3^)	Mean ± SD (μg m^−3^)	Median (μg m^−3^)	Mean ± SD (μg m^−3^)		
Primary	PM_2.5_	Overall		5.43	22.53 ± 37.45	1.09	4.27 ± 10.18	81.1	**0.004**		5.80	19.59 ± 43.59	2.44	4.74 ± 6.17	75.8	0.136
		Day		5.10	28.62 ± 48.50	1.56	3.91 ± 7.26	86.4	**0.008**		7.23	21.66 ± 47.14	2.81	6.46 ± 9.13	70.2	0.182
		Night	14	3.06	10.61 ± 23.89	0.41	5.01 ± 16.43	52.8	**0.003**	12	1.60	15.44 ± 37.38	0.77	1.41 ± 1.89	90.9	0.060
	PM_10_	Overall		7.10	28.74 ± 46.92	1.87	5.92 ± 12.62	79.4	**0.003**		6.97	31.70 ± 62.34	3.20	8.93 ± 11.88	71.8	0.117
		Day		7.55	36.39 ± 61.48	2.74	5.73 ± 9.24	84.3	**0.004**		8.97	36.91 ± 69.07	3.85	12.62 ± 17.88	65.8	0.136
		Night		4.31	13.76 ± 28.85	0.85	6.30 ± 19.91	54.2	**0.004**		2.23	21.20 ± 52.25	1.06	1.73 ± 2.12	91.8	**0.034**
Secondary	PM_2.5_	Overall		8.06	19.15 ± 31.02	2.77	7.03 ± 11.20	63.3	0.109		2.44	4.55 ± 4.29	2.65	3.52 ± 2.86	22.7	0.374
		Day		7.41	22.34 ± 33.10	2.79	5.58 ± 6.78	75.0	**0.041**		3.32	5.18 ± 4.65	3.02	4.68 ± 4.31	9.7	0.859
		Night	14	2.94	13.08 ± 36.72	1.52	9.96 ± 21.25	24.0	0.826	9	0.88	3.27 ± 6.38	1.22	1.29 ± 1.10	60.6	0.674
	PM_10_	Overall		9.94	24.66 ± 38.35	3.25	8.70 ± 13.41	64.7	0.140		2.74	6.19 ± 6.58	3.07	4.13 ± 3.39	33.3	0.441
		Day		9.81	29.47 ± 42.59	3.33	6.76 ± 8.16	77.1	**0.041**		3.77	7.44 ± 8.63	3.60	5.60 ± 5.15	24.7	0.767
		Night		3.16	15.43 ± 44.06	1.87	12.64 ± 25.91	18.1	0.730		1.08	3.66 ± 6.97	1.22	1.32 ± 1.07	63.9	0.594

**Table 5.** PAC effectiveness values in high-PM homes (> 5 μg m^−3^), as determined by paired approach.

PM Fraction	Time Period	Primary Rooms				Secondary Rooms			
		n	Percentiles			n	Percentiles		
			25^th^	50^th^	75^th^		25^th^	50^th^	75^th^
PM_2.5_	Overall	15	64.1%	81.7%	94.1%	11	13.9%	55.2%	92.6%
	Day	15	57.6%	77.9%	94.5%	11	45.5%	67.7%	91.4%
	Night	15	55.2%	89.7%	98.0%	11	−72.9%	42.8%	93.3%
PM_10_	Overall	15	65.5%	75.9%	94.3%	11	6.6%	68.9%	92.5%
	Day	15	54.8%	77.3%	94.6%	11	43.6%	73.4%	91.3%
	Night	15	49.1%	89.6%	97.9%	11	−75.6%	44.5%	93.9%
