# Supplementary material for: Real-World Effectiveness of Portable Air Cleaners in Reducing Home Particulate Matter Concentrations
Source: Aerosol Air Qual Res. Author manuscript; Available in PMC 2024 Apr 12. (PMC11014421; doi:10.4209/aaqr.230202)
Supplement: Appendices, including supplementary tables and figures [file NIHMS1968503-supplement-Appendices__including_supplementary_tables_and_figures.pdf]

Appendices

Table A1. *Median effectiveness (%) of the portable air cleaner (PAC) in the filter state in reducing $PM_{2.5}$ and PM_{10} concentrations in individual homes. Baseline concentration = 1 μg/m³ based on the PM sensor's limit of detection (LOD). WSRT = Wilcoxon signed rank test. $p < 0.05$ was considered significant (bolded).*

		Primary Rooms				Secondary Rooms				
**PM Fraction**	**Time Period**	**n**	**Percentiles**			**n**	**Percentiles**			**WSRT p-value**
			**25th**	**50th**	**75th**		**25th**	**50th**	**75th**	
$PM_{2.5}$	Overall	23	<0	66.0	90.1	22	<0	26.2	63.3	**<0.001**
	Day	23	<0	58.3	89.2	22	<0	47.4	73.4	**0.002**
	Night	23	11.6	83.0	98.0	22	<0	14.2	79.8	**0.001**
PM_{10}	Overall	23	<0	65.5	82.0	22	<0	27.1	69.9	**0.003**
	Day	23	<0	57.8	83.0	20	<0	45.6	73.9	**0.014**
	Night	23	<0	73.5	93.9	21	<0	29.4	83.2	**0.012**

Table A2. *PAC effectiveness (%) in low-PM homes (≤5 μg/m³) for the individual pairs of filter and sham periods (i.e., paired approach).*

		Primary Rooms				Secondary Rooms			
**PM Fraction**	**Time Period**	**n**	**Percentiles**			**n**	**Percentiles**		
			**25th**	**50th**	**75th**		**25th**	**50th**	**75th**
$PM_{2.5}$	Overall	11	-86.3%	-26.1%	66.2%	12	-500.3%	-120.4%	23.9%
	Day	11	-83.1%	-7.8%	87.8%	12	-497.1%	-89.6%	43.9%
	Night	11	-166.7%	26.5%	97.1%	12	-372.1%	-134.3%	45.7%
PM_{10}	Overall	11	-56.0%	9.5%	49.6%	12	-549.0%	-132.2%	27.1%
	Day	11	-74.8%	8.2%	69.7%	12	-730.8%	-97.2%	38.1%
	Night	11	-175.3%	34.4%	83.4%	12	-384.8%	-13.3%	77.7%

Panel 1

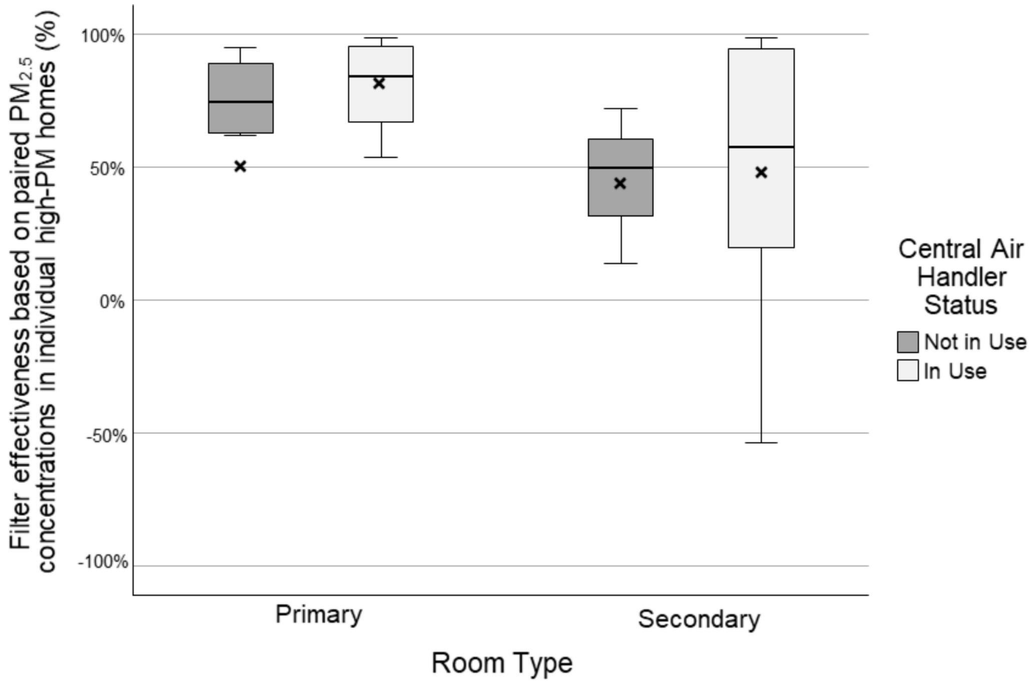

Panel 2

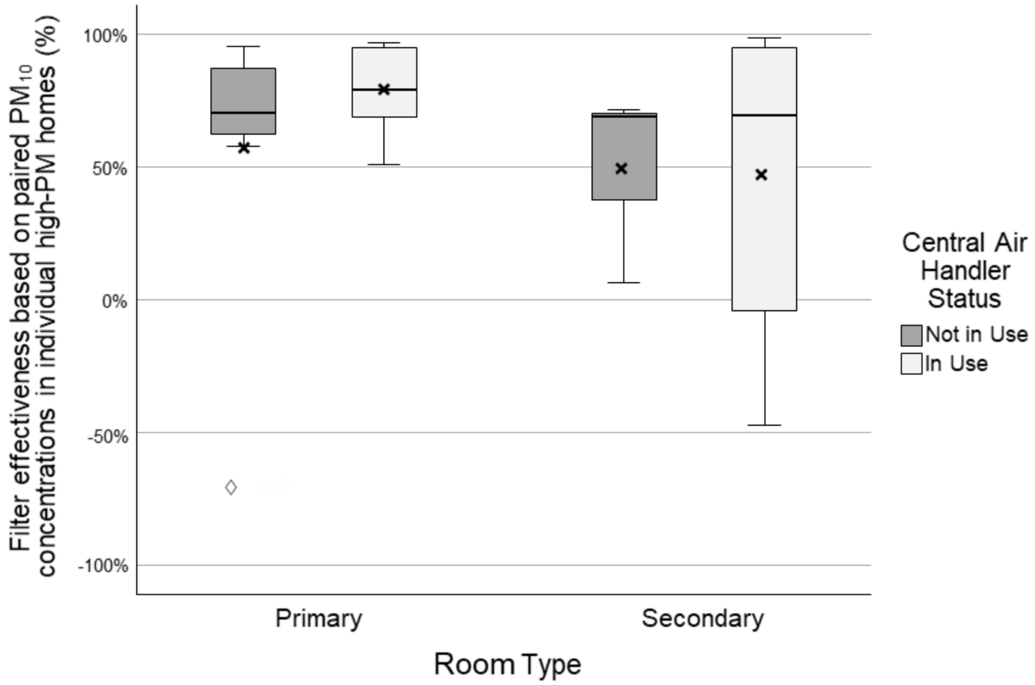

Figure A1. *Effect of a central air handler (CAH) on the filtration effectiveness (%) of the PAC in reducing mean concentrations of $PM_{2.5}$ (Panel 1) and PM_{10} (Panel 2) in the primary and secondary rooms of individual high-PM (>5 µg/m^3) homes. "In Use" = activation of the CAH for any length of time; "Not in Use" = CAH not activated or not present.*
